# An Ultra‐Broadband High Efficiency Polarization Beam Splitter for High Spectral Resolution Polarimetric Imaging in the Near Infrared

**DOI:** 10.1002/advs.202201227

**Published:** 2022-07-12

**Authors:** Hui‐Hsin Hsiao, Richard E. Muller, James P. McGuire, Deacon J. Nemchick, Chin‐Hung Shen, Gerard van Harten, Mayer Rud, William R. Johnson, Austin D. Nordman, Yen‐Hung Wu, Daniel W. Wilson, Yih‐Peng Chiou, Myungje Choi, Jason J. Hyon, Dejian Fu

**Affiliations:** ^1^ Institute of Electro‐Optical Engineering National Taiwan Normal University Taipei 11677 Taiwan; ^2^ Jet Propulsion Laboratory California Institute of Technology Pasadena CA 91109 USA; ^3^ Graduate Institute of Photonics and Optoelectronics National Taiwan University Taipei 10617 Taiwan; ^4^ Present address: Department of Engineering Science and Ocean Engineering National Taiwan University Taipei 10617 Taiwan

**Keywords:** gap‐plasmon metasurface, high‐resolution multiple‐species atmospheric profiler, plasmon hybridization, spatial‐spectral‐polarimetric images

## Abstract

A broadband, high efficiency polarized beam splitter (PBS) metagrating based on integrated resonant units (IRUs) to enable simultaneous polarization analysis, spectral dispersion, and spatial imaging in the near infrared (NIR) is developed. A PBS metagrating with a diameter of 60 mm is the key technology component of the high‐resolution multiple‐species atmospheric profiler in the NIR (HiMAP‐NIR), which is a spaceborne instrument concept crafted to be a core payload of NASA's new generation Earth System Observatory. HiMAP‐NIR will enable the aerosol profiling in Earth's planetary boundary layer (from surface to2 km altitude) by simultaneously measuring four spatial‐spectral‐polarimetric images from 680 to 780 nm. Through detailed optimization of hybridized resonant modes in IRUs, the PBS metagrating shows a diffraction efficiency of 70% (or better) for all four linear‐polarized incident light, and polarization contrasts between orthogonal states are 0.996 (or better) from 680 to 780 nm. It meets the stringent performance required by the HiMAP‐NIR exploiting a new paradigm for the broad applications of metasurfaces.

## Graphical abstract

An polarized beam splitter, enabling to simultaneously measure four spatial‐spectral‐polarimetric images with an efficiency ≈70% in the entire near‐infrared oxygen bands and a polarization contrast greater than 0.996, are realized by hybridized gap‐plasmonic metasurfaces. This meets the standard of NASA's high‐resolution multiple‐species atmospheric profiler instrument for aerosol profiling in the planetary boundary layer.

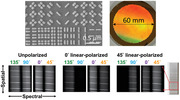

## 1 Introduction

Global, high‐resolution, and vertical profile measurements of gaseous pollutants (ozone and nitrogen dioxide) and aerosols are the three observables required by the Earth Science Decadal Survey community for advancing the current understanding of these pollutants on air quality, human health, and economic prosperity.^[^ ^1^, ^2^, ^3^, ^4^ ^]^ Quantification of these pollutants requires a technology advancement in order to measure their concentrations in the planetary boundary layer (PBL, 0–2 km above the Earth's surface), where emissions and subsequent photochemistry act to degrade air quality and impact human health and agricultural productivity, with a high spatial resolution (2 km × 2 km) and a broad spatial coverage to map spatial variability at the neighborhood (intra‐urban) scale over the source regions of these pollutants (e.g., megacities). Conventional spaceborne instruments,^[^ ^5^, ^6^, ^7^, ^8^, ^9^, ^10^, ^11^, ^12^, ^13^, ^14^, ^15^, ^16^, ^17^, ^18^ ^]^ built for inferring the aerosols’ total columns, lack sensitivity to quantify the vertical distribution of aerosols in PBL. Based on Observation System Simulation Experiments studies,^[^ ^19^, ^20^ ^]^ adding high spectral resolution linear polarimetry into radiance measurements within the oxygen A and B bands in the near infrared (NIR, 680–780 nm) enables improvement of accuracy in atmospheric aerosol profiling. As solar light is polarized when it is scattered by particles and gases in the atmosphere, the observed degree of linear polarization (DoLP) of solar light is determined by the size, shape, and vertical distribution of particles in the Earth's atmosphere.

**Figure 1:**
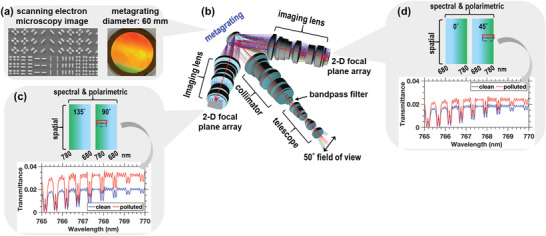
The illustration of HiMAP‐NIR. a) A photo of a PBS metagrating with a diameter of 60 mm that has been built as HiMAP‐NIR's key technology component. b) The lens assemblies for HiMAP‐NIR are now being built, and the entire instrument will be integrated in spring 2022. The red line depicts the light path. c,d) The four polarized spectra are imaged on the twin focal plane arrays. The segments of simulated spectra depict the HiMAP‐NIR's measurement capability of distinguishing the polluted scene (red spectra) from the clean scene (blue spectra). The polluted scene has a layer of aerosols in the PBL with an aerosol optical depth (AOD) of 1.0, a layer thick of 600 meters and layer peaking at 1 km, while no aerosol (AOD = 0) is present in the clean scene.

To address these science needs, the high‐resolution multiple‐species atmospheric profiler in the NIR (HiMAP‐NIR) is being crafted to enable the profiling of global PBL aerosols by simultaneously measuring four spatial‐spectral‐polarimetric images representing the four linear polarization states (0°, 90°, 45°, and 135°) of the atmosphere. HiMAP‐NIR will use sun light that travels through Earth's atmosphere and reflects from Earth's surface as its light source. These images can accurately determine the high spectral resolution radiance and the DoLP spectra of atmospheric constituents and surface properties for quantifying the vertical distribution of aerosols in the PBL. **Figure** **1** shows the schematic diagram of HiMAP‐NIR system. The broadband polarized beam splitter (PBS) metagrating chip with a diameter of 60 mm has been built and will be integrated into HiMAP‐NIR optical system in spring 2022. HiMAP‐NIR will enable the 3D mapping of PBL aerosols through simultaneous radiance and polarization measurements at three viewing angles from a low earth orbit (LEO, at 700 km altitude) with a wide field of view (50°), a signal‐to‐noise ratio (SNR) of 300:1, and a spectral resolving power (*λ*/Δ*λ*) of 4300 (see Section S1, Supporting Information). To meet the measurement requirements of the HiMAP‐NIR system, the design goals of grating diffraction efficiency, the accuracy of linear polarization measurements, and the size of the metagrating were estimated through the standard procedure of radiative transfer modeling and optics system design based upon HiMAP‐NIR's targeted spectral SNR, the capability of capturing the radiance/polarization spectral signals of PBL aerosols, the spectral resolution, and spatial coverage for this spaceborne optical remote sensing instrument. The resulting requirement of metagrating's diffraction efficiency (i.e., the ratio of diffracted radiance divided by the light incidence on the metagrating surface) is 70% to achieve the targeted SNR within 0.3 s measured time of each satellite's footprint (2 km × 2 km) on the ground. A metagrating with a diameter of 60 mm is needed in order to meet the required spectral sampling across the satellite track and the spectral SNR. In addition, a tolerance below ±0.005 is needed for the polarimetric contrast measurement in order to capture the radiance and polarization spectral signals of targeted PBL pollutants.

To achieve these design goals, we employed integrated resonant units (IRUs) in the PBS metagrating designs which will spatially separate the input light into four linear‐polarized (LP) states for the analysis of the DoLP in each wavelength bin. A variety of polarization‐multiplexing metadevices^[^ ^21^ ^]^ such as polarization convertors,^[^ ^22^, ^23^ ^]^ polarization‐dependent beam splitters,^[^ ^24^, ^25^, ^26^, ^27^, ^28^ ^]^ meta‐polarimetry,^[^ ^29^, ^30^, ^31^ ^]^ metalenses,^[^ ^32^, ^33^, ^34^, ^35^, ^36^ ^]^ metaholograms,^[^ ^37^, ^38^, ^39^, ^40^, ^41^, ^42^, ^43^, ^44^ ^]^ and full‐Stokes polarization cameras^[^ ^45^ ^]^ have been developed for versatile applications. Several pioneering works utilized IRU designs, which combined multi‐nanorod configuration into one unit cell, to eliminate the chromatic aberration or to enhance working efficiency over a continuous broad spectral range for the development of broadband achromatic beam deflectors,^[^ ^46^ ^]^ achromatic metalenses,^[^ ^46^, ^47^, ^48^, ^49^ ^]^ and high efficiency versatile polarization convertors.^[^ ^49^ ^]^ The IRU metasurfaces have been demonstrated as a robust method in tailoring broadband phase dispersion and efficiency enhancement for circular‐polarized light.^[^ ^49^, ^50^ ^]^ In this work, we demonstrated that IRU metasurfaces can be an effective platform in the manipulation of LP light with a high diffraction efficiency across a broad spectral range. Through the near‐field coupling of multiple nanobricks, the hybridized modes in IRUs achieve a broadband reflection efficiency enhancement and retain the required phase value with a linear and smooth phase dispersion for the co‐polarized light. The IRU‐based PBS metagrating shows an ultra‐broadband efficiency enhancement from wavelengths (*λ*) of 600 to 1100 nm outperforming their single‐rod counterparts, when simultaneously analyzing the intensity of four LP states of an input light beam. In addition to its broadband high‐efficiency (70% or better), a few technical characteristics of this spectropolarimetric imaging metagrating technology help in fostering a new paradigm for the broad applications of the metasurface family as the metagrating does not require multiple entrance slits, moving parts, high frequency modulation, or complex coating design and engineering, thus reducing implementation risk, mass, volume, and power of a spaceborne instrument as well as extending opportunities to a more diverse set of spaceborne platforms and launch vehicles.

## 2 Results and Discussion

### 2.1 Design of the Broadband PBS Metagratings

**Figure 2:**
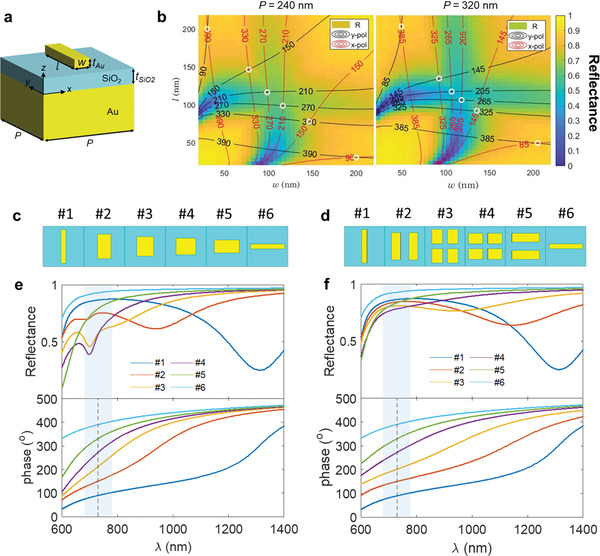
Design of the PBS metagratings. a) Sketch of a MIM unit cell for gap‐plasmonic metasurfaces. b) Calculated reflectance and phase contours as a function of nanobrick geometry for *P* = 240 nm (left) and *P* = 320 nm (right) at the central design wavelength of 730 nm. The thickness of the bottom gold layer was fixed at 150 nm, and both the thicknesses of top Au nanobricks and the SiO_2_ spacer were optimized to 50 nm to ensure the required phase coverage for 6‐level metasurfaces. The color map shows the minimal reflectance by comparing the results under *x* and *y*‐polarized light illumination. The reflection phase for both *x* and *y* polarization is discretized into six constant‐phase contour lines with a step of 60°, and the appropriate nanobrick dimensions corresponding to the intersections of a constant phase step of +60° and −60° for *y* and *x*‐polarized light, respectively, were selected to compose the 0°/90° (left) and 45°/135° (right) PBS metagratings (marked with circles). Supercells of 0°/90° PBS metagratings for c) single‐rod design and d) IRU design. e,f) The reflectance (top) and phase (bottom) spectra for each composed element. The gray areas depict the oxygen bands with a dashed line indicating the central wavelength (*λ* = 730 nm) of the spectral region of interest.

**Figure** **2**a shows the diagram of a metal‐insulator‐metal (MIM) unit cell consisting of a gold nanobrick on top of a SiO_2_ spacer and a gold back reflector. To diffract two orthogonal polarization states into opposite directions (i.e., +1 and −1 diffraction orders), metagratings should have an opposite‐signed linear phase gradient between the two polarizations. Two degrees of freedom in the nanobrick geometry (width (*w*) and length (*l*) as defined in Figure 2a) enables independent control of the reflected phases of two orthogonal polarized light. Figure 2b shows the calculated reflection and phase contour lines as a function of the nanobrick geometry at the central design wavelength of 730 nm. We intentionally chose elements possessing an inverse symmetry with respect to the diagonal line of *w* = *l* so that the second half of the composed elements are the 90° rotation of the first half (Figure 2c,d; see details in Sections S3,S4, Supporting Information). As shown in Figure 2e, since a rapid phase variation takes place close to a resonance, to fulfill the required phase values, the magnetic‐dipole (MD) mode of several selected single‐rod unit cells (i.e., #2 to #5) manifests itself as a reflection dip near the oxygen bands (grey area), which limits the optimal diffraction efficiency of metagratings. In addition, owing to the different resonant wavelengths of the MD modes among the composed unit cells, the diverse slope variation of phase dispersion makes the metagrating supercell no longer retain an equidistant phase difference at the spectral range away from *λ* = 730 nm (Figure 2e), thus diminishing its broadband performance.

To boost the diffraction efficiency of metagratings over a continuous broadband, especially in the spectral region of HiMAP‐NIR instrument, we optimized the design of IRU unit cells in order to achieve a broadband reflection efficiency enhancement while retaining the required phase value at the design wavelength as well. In addition, as we kept unit cells #1 and #6 identical owing to their off‐resonant property with a large reflectance above 80% from *λ* = 650 to 1000 nm (Figure 2e), it is desired that all unit cells composed of metagratings possess similar smooth and linear phase dispersion in order to maintain a constant phase step within the broad spectral region. Two categories of IRUs, including double‐rod and quadruple‐rod types, were implemented as shown in Figure 2d. To illustrate the methodology of simultaneously manipulating the reflection efficiency and the phase dispersion through tailoring the hybridization of resonances among the nanobricks, we analyzed the calculated reflection and phase response of the original single‐rod designs, the replaced IRUs, and the constituent single‐rod of IRUs before plasmonic coupling under *y*‐polarized light. The discussion is also valid for the optical response of the orthogonal polarized light since the variations of *l* and *w* for the selected elements are exactly the reverse of each other.

**Figure 3:**
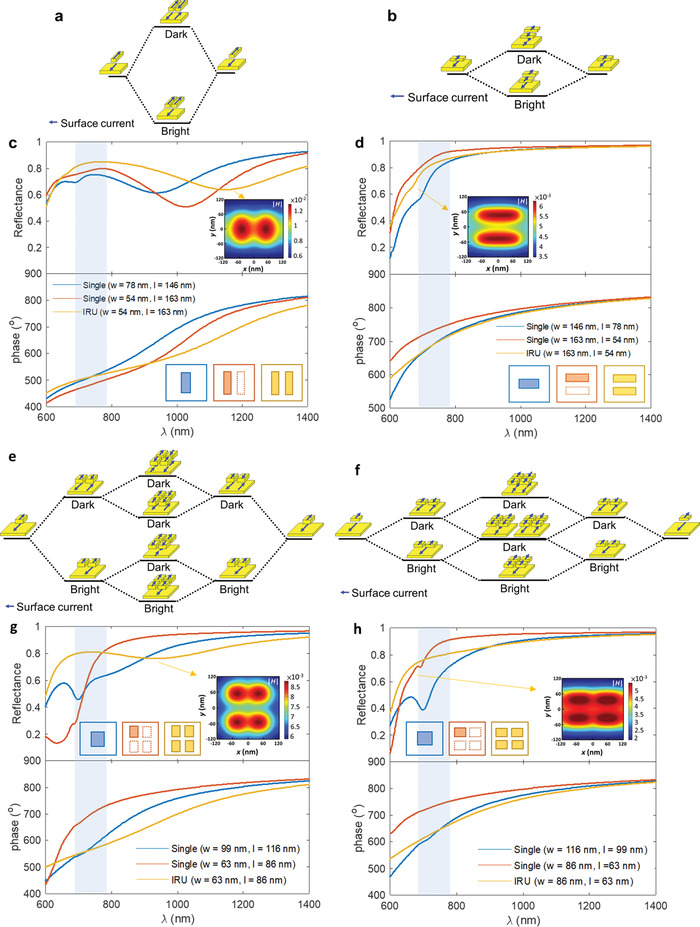
The hybridization of the MD resonances for double‐rod IRUs. Schematic of the possible hybridized states in a MIM dimer consisting of a) large AR nanobricks arranged in left–right and b) small AR nanobricks arranged in front–back configurations, and that of a MIM quadrumer for the constituent nanobrick with AR e) greater and f) less than but close to 1. c,d,g,h) The simulated reflectance (top) and phase (bottom) spectra for the single‐rod element (blue), the replaced IRU (yellow), and the constituent single nanorod in IRU (red). Insets show the magnetic‐field distributions for the hybridized modes in IRUs. The gray areas depict the oxygen bands.

Starting from the case for large aspect‐ratio (AR) nanobrick that supports a MD mode at the red‐side of the design band (i.e., element #2 at *λ* = 950 nm in **Figure** **3**c), where the AR is defined as the ratio of nanobrick lengths parallel and perpendicular to the light polarization (i.e., AR = *l*/*w*), if one intuitively enlarges *l* to further redshift the MD mode for the increment of the reflectance in the oxygen bands, the deviation of phase response takes place as well (red curve in grey region of Figure 3c). To increase the reflection and retain the required phase range simultaneously, two prolonged nanorods were arranged in a left–right configuration to form a double‐rod IRU, which results in an even more pronounced redshifted resonance at *λ* = 1100 nm with a shallower and broadening dip (yellow curve). Meanwhile, the phase response is shifted back to the required value at the central wavelength of the HiMAP‐NIR instrument (*λ* = 730 nm). This behavior is associated to an increasing effective refractive index due to plasmonic coupling of the MD modes. As displayed in Figure 3a, the bright hybridized MD mode (radiative mode) has parallel induced currents between the nanobricks resulting in a net dipole at the lower energy branch that is detectable in the far field. On the contrary, the induced currents for the dark hybridized MD mode (nonradiative mode) at the higher energy branch is antiparallel, thus rendering a zero net dipole which is difficult to excite and measured with light.^[^ ^51^ ^]^ The spatial overlap of the magnetic‐field distribution in the middle gap also reveals the coupling effect for the hybridized MD modes (inset of Figure 3c). Thus, the redshifted and shallower hybridized resonance in IRU design leads to a significant reflection enhancement from 600 to 1000 nm. In addition, the broadening bandwidth at the long wavelength significantly reduces the degree of abrupt phase jump and approaches to linear and smooth phase dispersion (yellow curve).

For a low‐AR nanobrick with its MD resonance excited at the blue‐side of the oxygen bands (i.e., element #5 at *λ* = 700 nm in Figure 3d), a nanobrick with shortened *l* has larger reflection in the oxygen bands as a result of the blueshifted MD mode but also accompanied a significant phase deviation (red curve in grey region of Figure 3d). Thus, we integrated the shortened double nanorods into a front–back arrangement with respect to the *y‐*polarized light instead. Different from the formation of the left–right double‐rod IRU, the hybridized MD mode (bright mode) in the front–back configuration exhibits a relatively small spectral shift with respect to its composed single‐rod element but still performs an increasing resonant bandwidth as indicated by the multipolar analysis shown in Figure S3, Supporting Information. It is worth noting that the amount of energy splitting for the hybridized modes is strongly dependent on the coupling strength between the resonators (Figure 3a,b). As a standing‐wave pattern of the magnetic field along the optical resonant cavity is strongly confined near the center of nanobrick length *l* upon the illumination of *y*‐polarized light, a more effective near‐field coupling arises between the left–right neighboring nanorods than between those in front–back configuration (insets of Figure 3c,d), thus resulting in a strong near‐field intensity and larger energy splitting for the left–right pair. As shown in Figure 3d, the blueshifted and broadening hybridized mode thus gives rise to an increasing reflection below the spectral range of 800 nm.

In addition, for the structure with AR greater than but close to 1 that supports the MD mode near the red side of the central spectral region (i.e., element #3 in Figure 3g), the quadruple‐rod IRUs were implemented as the coupling effect of double‐rod type IRUs is not sufficient for manipulating the reflection and the required phase value at will. To form a nanorod quadrumer, we integrated four nanoblocks with narrower *l* and *w* that individually supports the MD resonance at 700 nm (red curve). Through the plasmonic coupling, the hybridized mode in quadruple IRU results in a more remarkable spectral shift to 950 nm (yellow curve). The hybridization process of quadruple nanorods was schematically illustrated by a two‐step hierarchical structure (Figure 3e). First, the left–right pairs of nanorods are coupled strongly resulting in two hybridized modes with a large splitting energy. Then, the double‐rod IRUs were further integrated through a front–back arrangement, leading to four possible hybridized states in the quadruple‐rod system with a smaller splitting energy between each other. Only the lowest energy branch with four parallel MD resonators belongs to a bright mode. The near‐field distribution also reveals that a stronger magnetic‐field resides in the gap between the left–right coupling pairs (inset of Figure 3g). The significant broadening and redshifted hybridized resonance leads to a broadband reflection enhancement from 600 to 900 nm and reaches 80% in the oxygen A and B bands. The smooth and linear phase dispersion over an ultrabroad spectral range from 600 to 1200 nm again demonstrates the advantage of IRU designs in sustaining a similar slope of phase dispersion to the off‐resonant cases.

On the other hand, when the quadruple‐rod IRUs were formed by integrating four nanobricks with AR less than but close to 1, the hybridized mode again exhibits a subtle spectral redshift and an increasing resonant bandwidth compared to its single‐rod counterpart. This behavior is associated to the fact that the enlarging width of the low‐AR nanorods decays the coupling effect between the left–right neighbors to a strength comparable to that of the front–back pairs as indicated in the inset of Figure 3h. As a result, the low‐AR quadruple‐rod IRU with a small spectral shift at the blue‐side of oxygen bands leads to a broadband reflection enhancement in the spectral region below 900 nm (Figure 3h). Figure 2f summarizes the reflection and phase spectra of the 0°/90° PBS IRU supercell. Each unit cell in the IRU‐based metagrating has a reflection larger than 75% within the continuous wavelength range of 650 to 1000 nm and is capable of retaining an equidistant phase step between each other over a significantly broader spectral range in contrast to the serious nonuniform phase gradient between the elements of the single‐rod design outside the central bands (Figure 2e).

### 2.2 Fabrication of the Broadband PBS Metagratings

**Figure 4:**
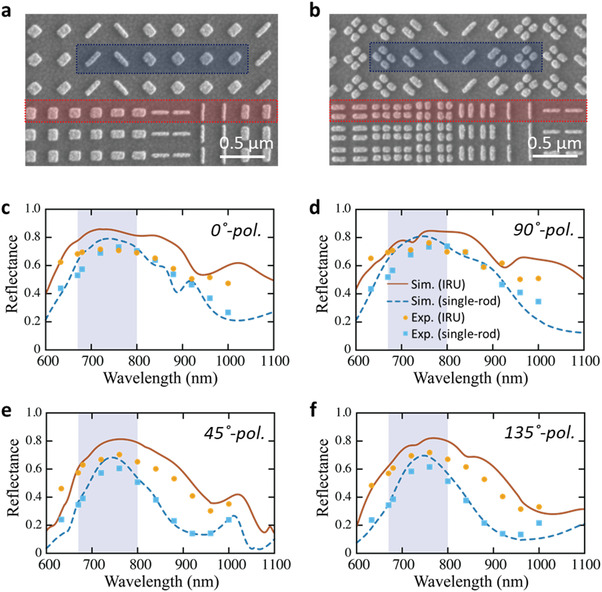
Diffraction efficiency of broadband PBS metagratings. SEM images for a region of fabricated a) single‐rod and b) IRU metagratings. These PBS metagratings comprised two interweaving supercells for the diffraction of 0°/90°(red) and 45°/135° LP light (blue), respectively. The diffraction efficiency spectra of 0°/90° PBS metagratings for diffracting c) 0°‐LP light to +1 order and d) 90°‐LP light to −1 order, and that of 45°/135° PBS metagratings for diffracting e) 45°‐LP light to +1 order and f) 135°‐LP light to −1 order. All the measured (orange dots) and simulated (red‐solid curve) results of IRU metagratings outperform the experimental (blue dots) and calculated data (blue‐dashed curve) of the conventional single‐rod design in the broad spectral region.

The metagratings were fabricated through metal/oxide evaporation, electron‐beam lithography, and lift‐off processing (see details in Experimental Section). Scanning electron microscope (SEM) images for both single‐rod and IRU supercells are shown in **Figure** **4**a,b, respectively. Each unit cell in the 0°/90° PBS metagrating was replicated twice to reduce the diffraction angles of metagratings to fit into the compact test beds that were integrated for the optical characterization of metagratings (see Sections S8,S9, Supporting Information). This replication has a trivial effect on the diffraction efficiency. The 0°/90° and 45°/135° supercells were arranged by interweaving one block by one block along the *y*‐direction, where each block contains one type of supercell duplicated 1600 times. This interweaving arrangement helps in achieving the homogeneous sampling of the spatial measurements across individual ground pixels and eliminating the undesired off‐diagonal diffraction spectra caused by the periodicity in the *y*‐direction, thus making use of all the photons at the targeted grating orders. Recently, the fabrication process has been optimally stabilized and applied to build a PBS metagrating with a diameter of 60 mm (see Figure 1), rendering the spatial coverage across the satellite ground track and a spectral resolving power of 4300 required by the HiMAP‐NIR instrument.

### 2.3 Optical Characterization of the Broadband PBS Metagratings

The fabricated metagratings were first characterized by measuring the diffraction efficiency, which is estimated by measuring the intensity of the first‐order diffraction beam and normalizing with the reflectivity from a gold substrate. Details of measurement setup can be found in Section S8, Supporting Information. To examine the broadband spectral response of metagratings, we used a tunable laser system operating in the wavelength range of 630 to 1000 nm as a light source. A power meter was used to record the intensity of incident and diffracted light, while twin charge‐coupled‐device cameras were implemented to record the spectral polarimetric images. Figure 4c–f shows the simulated and measured efficiency of the IRU‐based metagratings for the diffracting 0^○^, 90^○^, 45°, and 135° LP states, which were overlaid by the grating efficiency data of the single‐rod metagratings. The diffraction angles for the four LP states agree well with the theoretical prediction validated by the near‐field diffraction patterns (see Figure S4, Supporting Information). As displayed in Figure 4c,d, the calculated efficiency of the IRU‐based 0°/90° PBS metagrating reaches almost 80% in the oxygen bands (brown‐solid curves), close to an ideal design limited by the intrinsic metallic loss. Apart from the central spectral region, an enhancement greater than 20% was achieved over the spectra compared to single‐rod counterpart and results in an ultrabroad full‐width at half maximum (FWHM) of 500 nm. The measured diffraction efficiency shows good agreement with the predicted values, where the slight efficiency drop of the experimental results (within 5–10%) may originate from the inevitable surface roughness and dimension variation for the fabricated samples. In addition, since the adhesion layers of titanium (Ti) were deposited (see details in Experimental Section) to ensure the stability during the lift‐off process especially for our large‐scale samples, these lossy Ti layers also play a role in degrading the conversion efficiency of fabricated samples (see details in Section S7, Supporting Information). The IRU metagrating achieves a measured efficiency of 70% (or better) in the NIR oxygen bands (orange dots in gray areas) and shows an efficiency greater than 50% from 600 to 1000 nm. The more prominent efficiency enhancement is achieved by the IRU‐based 45°/135° PBS metagratings whose experimental and simulated results show greater than 10% diffraction efficiency enhancement over that of single‐rod design within the oxygen bands and greater than 30% enhancement outside the central bands (Figure 4e,f). The measured efficiency is close to 70% for IRU metagratings within the oxygen bands (orange dots in gray regions), while the efficiency of single‐rod design is 60% near the central design wavelength and drops to 40% at *λ* = 680 nm.

**Figure 5:**
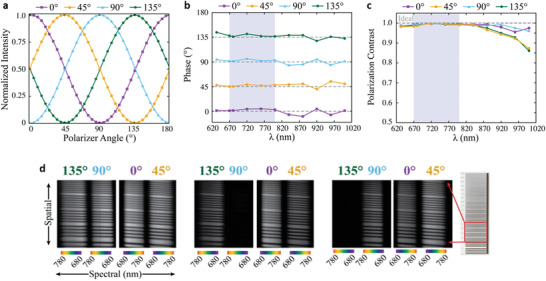
Polarimetric characteristics of the broadband IRU‐based PBS metagratings. a) The intensity variation of 0°, 45°, 90°, and 135° polarization states, when the polarizer was rotating from 0° to 180°, at 765 nm monochromatic laser source illumination. The spectral response of b) polarimetric modulation phase and c) contrast in polarimetric modulation. d) Spectral polarimetry images recorded using a fabricated NIR metagrating upon the impinging of unpolarized (left images), 0°‐LP (middle images), and 45°‐LP (right images) white light as input. A variable line grating target, in which the rectangular box indicates the target lines recorded in the images.

Then, we investigated the polarimetric performance of IRU metagratings in terms of polarization modulation, polarization contrast, and spatial imaging. We first calibrated the optical property of each diffraction beam representing the four LP states at *λ* = 765 nm by making a set of measurements in which light traverses specifically oriented polarizers before reaching the detector. **Figure** **5**a shows the recorded normalized intensity when the polarizer was rotating from 0° to 180° and the incident laser beam was set to the known polarization states with a sampling interval of 5°. Each diffraction beam displays a maximal value when the input polarization is parallel to the orientation of the metagrating polarization directions and exhibits nearly complete extinction when they are perpendicular to each other. Thus, the polarimetric contrast of each diffraction beam can be estimated by evaluating the value of (*I*_max_ *− I*_min_)/(*I*_max_ + *I*_min_), where *I*_max_ and *I*_min_ are the recorded maximal and minimal intensity of each diffraction beam during the polarization modulation. Shown in Figure 5b, the phases of measured diffracted light are nearly constant across the entire spectral range of 630 and 1000 nm. The measured polarimetric contrast is >0.996 from *λ* = 630 to 840 nm, which is close to this ideal value of 1.0 (Figure 5c) meeting the targeted performance required by the HiMAP‐NIR instrument.

The spaceborne HiMAP‐NIR instrument will implement a PBS metagrating to conduct the spectropolarimetric imaging using reflected sun light as the source. To demonstrate this measurement concept, shown in Figure S7, Supporting Information, we used a laboratory white light source, placed a variable line target in the light path of the integrated test setup, and used twin cameras to simultaneously record four spectral polarization images diffracted from the PBS grating. The recorded images (Figure 5d) show expected intensity responses with respect to the incidence of unpolarized, 0°‐LP, and 45°‐LP white light, respectively. For example, under the unpolarized light illumination, the intensity for the diffraction patterns of the four LP states was evenly distributed. When using 0°‐LP white light as input, one observes a distinct intensity contrast between 0° and 90°‐LP diffraction beams, while the diffraction patterns for 45°/135°‐LP states are about a factor of two dimmer than that of 0°‐LP states. Similar results were recorded for the incidence of 45°‐LP white light sources. The bright–dark intensity variation along the spatial regime in the recorded images clearly represent the spatial pattern of the variable line target (16.67 line pairs mm^−1^) placed in the light path, exploiting the spatial imaging functionality of this metagrating.

## 3 Conclusion

A broadband high‐efficiency IRU‐based PBS metagrating enabling the polarization analysis, spectral dispersion, and spatial imaging simultaneously has been developed as the key technology component of the spaceborne HiMAP‐NIR instrument. HiMAP‐NIR is being crafted to be a core payload of NASA's new generation Earth System Observatory. Operating in the spectral region of 680 to 780 nm, HiMAP‐NIR will simultaneously measure four spatial‐spectral‐polarimetric images every 0.3 s to enable the global aerosol 3‐D mapping in Earth's PBL at the neighborhood (intra‐urban) scale for advancing the understanding of air quality science and its societal impacts.

We developed a compilation of hybridization models that consider the coupling between multiple nanorods to help understand the underlying physics of the optimal resonant modes in IRUs, guiding the design optimization to meet the stringent performance required by the HiMAP‐NIR instrument. Through the interplay of multi‐rod resonances, we optimize the design of IRU elements to enable a broadband reflectance enhancement, sustain the required phase response at the design central wavelength, and tailor a linear and smooth phase dispersion which were found greatly beneficial to retain a constant phase step between the meta‐atoms in one supercell over a broad bandwidth. Both the simulated and measured optical performance of the IRU‐based PBS metagrating exhibit an ultra‐broadband efficiency enhancement from *λ* = 600 to 1000 nm outperforming their single‐rod counterparts, which reaches ≈70% efficiency over the entire NIR oxygen bands and a polarization contrast greater than 0.996 for the four LP states. An IRU‐based PBS metagrating with a diameter of 60 mm has been fabricated and will be integrated into the HiMAP‐NIR instrument, fostering a new paradigm for the broad applications of the metasurface family.

## 4 Experimental Section

### Sample Fabrication

First, a layer of Ti (thickness of 3 nm) was deposited on a silicon substrate using e‐beam evaporation, followed by a 150‐nm‐thick Au layer. An additional Ti layer with the thickness of 1 nm was evaporated to the top of the gold film to ensure the adhesion of the SiO_2_ layer. Then, 50 nm of SiO_2_ film working as the dielectric spacer was deposited by magnetron sputtering machine. Next, the sample was coated with 75 nm of polymethyl methacrylate copolymer, which overlaid by a layer of ZEP520 electron beam resist (thickness of 50 nm). A 20 nm coating of chrome was then applied to prevent charging during e‐beam writing. The metagrating patterns were defined by e‐beam lithography using a JEOL 9500FS operating at 48 kV. The ZEP520 layer was developed in ZED‐N50, followed by an isopropanol/water mixture to develop the copolymer layer. After a short descum step, the top metal layer was deposited. A half‐nanometer adhesion layer of Ti was evaporated, followed by 50 nm Au. Lift‐off was carried out in an n‐methyl‐2‐pyrrolidone solvent bath, heated to 70 °C.

### Numerical Simulation

All the simulations were performed by commercial software Computer Simulation Technology (CST) Microwave Studio based on the finite‐element frequency‐domain solver. For the case of periodic structures, the unit cell boundary conditions were used in the simulation of converted efficiency, reflection, phase profile, and near‐field distributions. A plane wave was impinging from air to the nanostructure at normal incidence for all the full‐wave calculations. At least 12 mesh steps per wavelength were used to ensure the accuracy of the calculated results. The permittivity of gold in the visible regime was described by a Drude–Lorentz model,^[^ ^52^ ^]^ and the refractive index of SiO_2_ was set to be 1.45. In addition, the charge‐current multipole expansion was implemented to quantitatively analyze the resonant property of the nanostructures.

## Conflict of Interest

The authors declare no conflict of interest.

## Author Contributions

H.‐H.H. led the design of the metagratings. R.E.M. led the fabrication of the metagratings. D.J.N. led the optical characterization of the metagratings. J.P.M. led the optics system design of HiMAP‐NIR. C.‐H.S. and Y.‐P.C. assisted with the metagrating design. G.V.H provided the experiment design and data analysis method of the optical characterization for the metagratings. M.R. assisted with the optics system design. A.D.N., W.R.J., and Y‐H.W. assisted with optical characterization of metagratings. D.W.W. guided the development of metagrating fabrication process. M.C. conducted the spectropolarimetric simulation of HiMAP‐NIR measurements. J.J.H. contributed to the formulation of spectropolarimeteric imaging technology. D.F. planned and organized the project, provided the design requirements of the metagratings and HiMAP‐NIR instrument, and helped in the optical characterization of the metagraings. H.‐H.H. and D.F. drafted the initial manuscript. D.W.W., J.P.M., R.E.M., D.J.N., A.D.N., and Y.‐H.W. provided updates. All authors reviewed and commented on the paper.

## Supporting information

Supporting Information

## Data Availability

The data that support the findings of this study are available from the corresponding author upon reasonable request.
