# Supplementary material for: An Ultra‐Broadband High Efficiency Polarization Beam Splitter for High Spectral Resolution Polarimetric Imaging in the Near Infrared
Source: Adv Sci (Weinh). 2022 Jul 12;9(27):2201227. doi: 10.1002/advs.202201227 (PMC9507354; doi:10.1002/advs.202201227)
Supplement: Supplementary file 1 — Supporting Information [file ADVS-9-2201227-s001.pdf]

ADVANCED SCIENCE
Open Access

Supporting Information

for *Adv. Sci.*, DOI 10.1002/advs.202201227

An Ultra-Broadband High Efficiency Polarization Beam Splitter for High Spectral Resolution Polarimetric Imaging in the Near Infrared

Hui-Hsin Hsiao, Richard E. Muller*, James P. McGuire, Deacon J. Nemchick, Chin-Hung Shen, Gerard van Harten, Mayer Rud, William R. Johnson, Austin D. Nordman, Yen-Hung Wu, Daniel W. Wilson, Yih-Peng Chiou, Myungje Choi, Jason J. Hyon and Dejian Fu**

Supporting Information

An ultra-broadband high efficiency polarization beam splitter for high spectral resolution polarimetric imaging in the near infrared

Hui-Hsin Hsiao[1,*], *Richard E. Muller*[2,*], *James P. Mcguire*[2], *Deacon J. Nemchick*[2], *Chin-Hung Shen*[3], *Gerard van Harten*[2], *Mayer Rud*[2], *William R. Johnson*[2], *Austin D. Nordman*[2], *Yen-Hung Wu*[2], *Daniel W. Wilson*[2], *Yih-Peng Chiou*[3], *Myungje Choi*[2], *Jason J. Hyon*[2], *and Dejian Fu*[2,*]

[1]Institute of Electro-Optical Engineering, National Taiwan Normal University, Taipei 11677, Taiwan

[2]Jet Propulsion Laboratory, California Institute of Technology, Pasadena, CA, 91109, USA.

[3]Graduate Institute of Photonics and Optoelectronics, National Taiwan University, Taipei 10617, Taiwan

E-mail: hhhsiao@ntnu.edu.tw; richard.e.muller@jpl.nasa.gov; dejian.fu@jpl.nasa.gov

Section S1: The High-resolution Multiple-species Atmospheric Profiler in the NIR

The High-resolution Multiple-species Atmospheric Profiler in the NIR (HiMAP-NIR) (Figure 1 in main text) is an instrument concept which is being crafted be a core payload of NASA's new generation Earth System Observatory for advancing the understanding of air quality science and its societal impacts. It consists of a telescope, a bandpass filter, an entrance slit, a collimator, a broadband polarized beam splitting (PBS) metagrating, two sets of imaging lenses, and twin imaging focal plane arrays (FPAs). A refractive telescope forms an f/3 image onto the entrance slit which is placed behind the bandpass filter. The collimator lens images the pupil on the metagrating, which splits light into four different polarization spectra of atmospheric constituents and directs the light to the imaging lens. The four polarized spectra are simultaneously imaged on the twin FPAs. The light is dispersed in the horizontal direction, while the slit is imaged in the vertical direction. The optical system will be mounted on a gimbal to dynamically aims the optical system at the ground along the satellite ground track to capture images from different view angles. This optimizes the information content of aerosol vertical distribution. A broadband PBS metagrating chip with a diameter of 60 millimeter is the key technology component of HiMAP-NIR. The system level integration, alignment, tests and calibration of HiMAP-NIR will be conducted using the world class facilities at JPL, e.g, the thermal vacuum chambers, the vibration testing laboratory, and the reference radiance and polarization light sources to ensure HiMAP-NIR system could survive the environment of launch process and suborbital/orbital platforms, as well as to achieve the precision and accuracy of radiance and polarization measurements. Then, the HiMAP-NIR system could be deployed on an aircraft platform (e.g. ER-2) to conduct science flights in which HiMAP-NIR will record the spectral resolved polarization spectra using reflective sunlight as source (see the simulated spectra in Figure 1c and 1d in main text). The retrieval algorithm of HiMAP-NIR will quantify the amount of PBL aerosols over the region of interest (e.g., San Francisco, see Figure 1e). It has been built at Jet Propulsion Laboratory and will be integrated with the HiMAP-NIR lenses (that are being built at JML Optical Industries Inc) in spring 2022. In the future, when onboarding an ESPA (Evolved Expendable Launch Vehicle Secondary Payload Adapter) class small satellite to fly in a Sun synchronous orbit (~700 km above surface), the HiMAP-NIR will provide the three-dimensional mapping of aerosols planetary boundary layer (surface-2 km) at the neighborhood (intra-urban) scale with a spatial resolution of 2 km by 2 km and a spatial coverage of ~800 km across the satellite ground track.

Section S2: The optimization of gap-plasmon metasurfaces.

We calculated the reflection and phase contour lines by varying the width (w) and length (l) of one single nanobrick at a fixed central wavelength (λ) of 730 nm. Upon normal incidence, the strong near-field coupling between the top antenna arrays and their mirror dipoles in the metallic plane induces two possible resonant states, known as the electric dipole (ED) mode and the magnetic dipole (MD) mode with parallel and antiparallel current distributions, respectively. Strong absorption of the incident light happens close to the MD resonance (bright mode) accompanied with a rapid phase variation (Figure S1). Although the geometry of top nanobricks are the dominant parameters in determining the MD resonances, the spacer thickness (t_{sio2}) and the nanobrick thickness (t_{Au}) play an important role in determining both the reflection and the phase range. We investigated the case when t_{Au} is fixed as 50 nm and varied the spacer thickness. As shown in Figure S1b, the reflection dip of the MD mode strengthens for smaller t_{sio2}, and the equidistant contour lines of the reflection phase is getting closer to each other. This behavior is associated to an increasing effective refractive index of the MD mode for a thinner spacer thickness, thus resulting in a stronger confinement of the MD mode with a more pronounced reflection dip as well as a predominant spectral shift when modifying the dimension of the nanobrick. On the other hand, Figure S1c shows the results when keeping t_{sio2} at 50 nm and changed t_{Au} instead. The thinner nanobrick demonstrates a similar effect to the spacer variation with a more pronounced reflection dip and crowded contour lines of the reflection phase. Thus, we fixed both t_{Au} and t_{sio2} at 50 nm in our study to ensure the required phase coverage for 6-level metasurfaces.

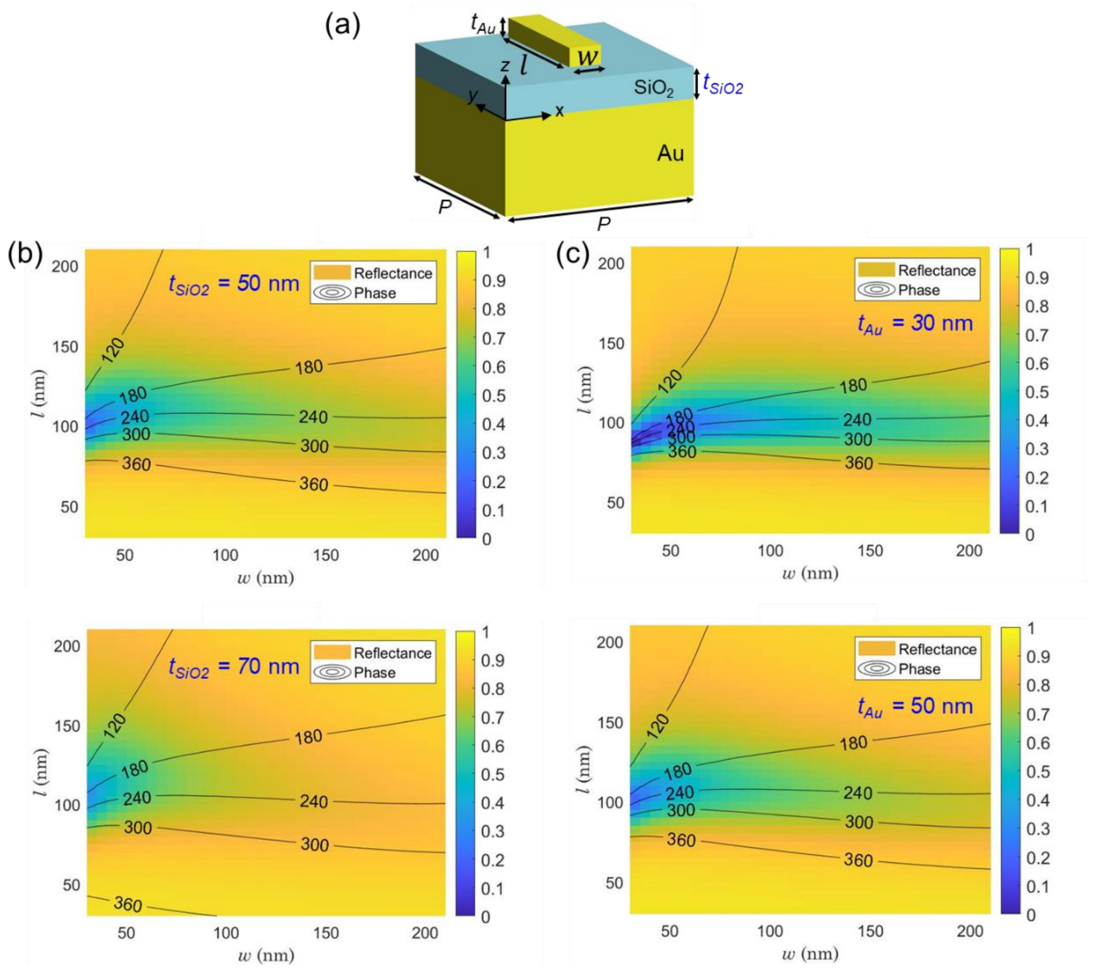

Figure S1. **(a)** Diagram of the unit cell for gap-plasmon metasurfaces with P = 240 nm. A y-polarized wave is normally incident to the surface at a wavelength of 730 nm. Calculated reflection (color map) and phase contours as a function of nanobrick geometry for varying **(b)** the spacer thickness t_{SiO2} (t_{Au} = 50 nm) and **(c)** the nanobrick thickness t_{Au} (t_{SiO2} = 50 nm).

Section S3: The structural parameters for the single-rod and IRU elements constituent broadband 0˚/90˚ PBS metagratings.

The optimal geometric parameters of the single-rod and integrated resonant units (IRU) PBS metagratings for diffracting 0˚/90˚ linear-polarized light are listed in Table S1. The periodicity of each unit cell was fixed at 240 nm. To make robust designs for the ease of manufacturing, the minimal size of nanobricks and the gap distance between them were set to be 30 nm. We intentionally chose elements with inverse symmtry to the line contouring $w = l$ in the phase maps (Figure 2b in main text) so that the variations of l and w for the selected elements are reverse for each other.

Table S1. The geometric parameters of the single-rod and the IRU PBS 0˚/90˚ metagratings.

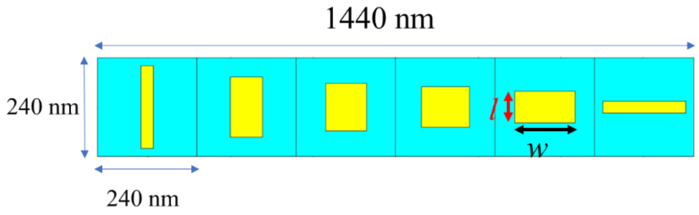

	1	2	3	4	5	6
w (nm)	30	78	99	116	146	200
l (nm)	200	146	116	99	78	30

(a)

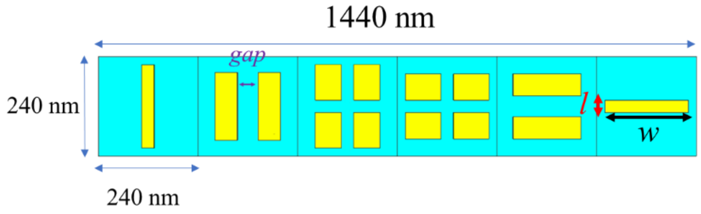

	1	2	3	4	5	6
type	1X1	1X2	2X2	2X2	2X1	1X1
w (nm)	30	54	63	86	163	200
l (nm)	200	163	86	63	54	30
gap		50	30	30	50	

(b)

Section S4: The structural parameters of the single-rod and the IRU elements constituent broadband 45˚/135˚ PBS metagratings.

The optimal geometric parameters of each unit cell with a periodicity of 320 nm are presented in Table S2.

Table S2. The geometric parameters of the single-rod and the IRU PBS 45˚/135˚ metagratings.

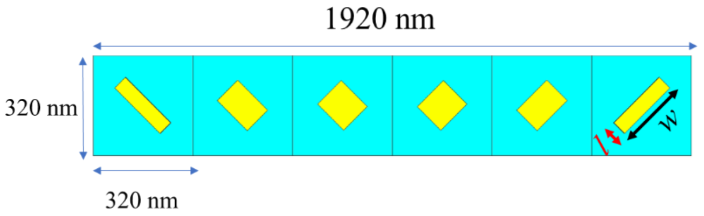

	1	2	3	4	5	6
w (nm)	49	92	107	118	136	205
l (nm)	205	136	118	107	92	49

(a)

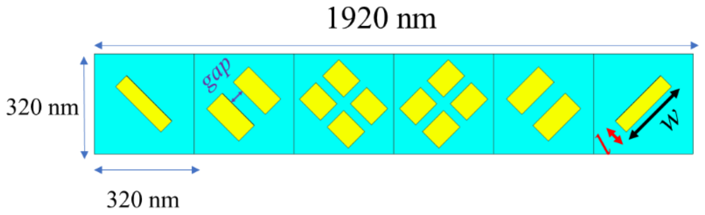

	1	2	3	4	5	6
type	1X1	1X2	2X2	2X2	2X1	1X1
w (nm)	49	70	74	96	146	205
l (nm)	205	146	96	74	70	49
gap		50	30	30	50	

(b)

Figure S2 shows the simulated reflection and phase spectra for each single-rod and IRU element constituent of the 45˚/135˚ PBS metagratings, respectively. In order to fulfill the required phase gradient of a beam deflector, unit cells with low reflection such as #2 to #5 were selected, which affects the overall diffraction efficiency. Meanwhile, the diversity for the slope of phase dispersion among the composed unit cells make it challenging to preserve the equidistant phase difference at the spectral range away from $\lambda = 730$ nm (Figure S2a). Through the optimization of IRUs' design, the replaced IRU supercells for 45˚/135˚ PBS metagratings have each element performing a reflection ~ 70% in the near-infrared (NIR) oxygen bands (Figure S2b). The broadening hybridized modes in IRU designs lead to linear and smooth phase dispersion from 600 nm to 1000 nm, which significantly reduces the deviation of the phase difference between unit cells within the spectra.

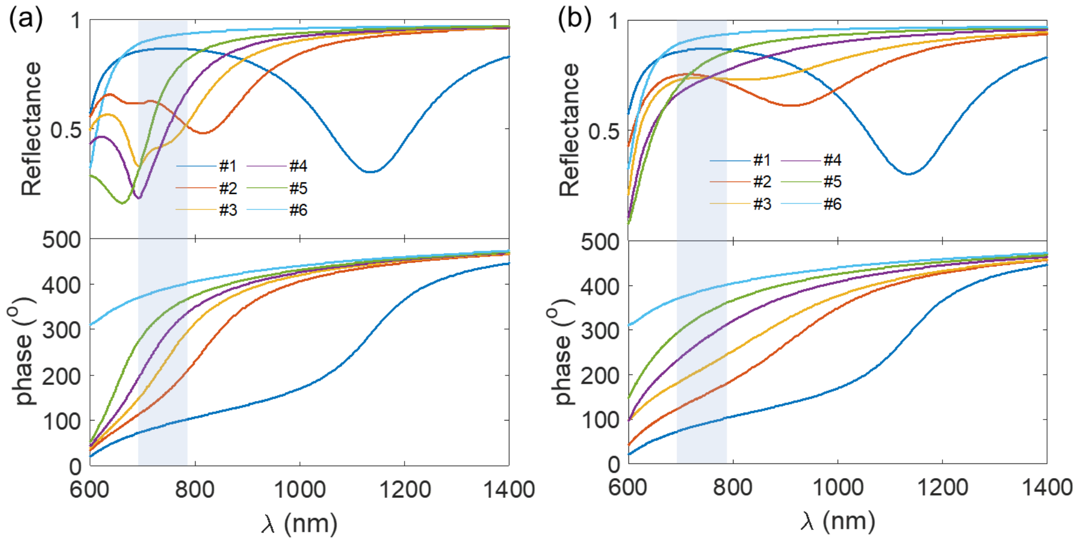

Figure S2. The reflectance (top) and phase (bottom) spectra for each composed unit cells of 45°/135° PBS metagratings in **(a)** the single-rod design and **(b)** the IRU design. The gray areas depict the oxygen bands.

Section S5: Multipolar decomposition spectral analysis of double-rod IRUs in front-back arrangement.

Since the MD resonant is indistinct in the spectra especially for low aspect-ratio (AR) nanobricks, we employed the multipolar decomposition method to quantitatively analyze the resonant property of these nanostructures. The charge-current multipole expansion was implemented by integrating the induced volume current density $\vec{J}$ over the designed nanostructures. The multipolar electric, magnetic, and toroidal moments are thus given by[1, 2],

Electric dipole moment: $\vec{P} = \frac{1}{i\omega}\int \vec{J} d^3 r$

Magnetic dipole moment: $\vec{M} = \frac{1}{2c}\int \left(\vec{r} \times \vec{J}\right) d^3 r$

Toroidal dipole moment: $\vec{T} = \frac{1}{10c}\int [(\vec{r} \cdot \vec{J})\vec{r} - 2r^2 \vec{J}] d^3 r$,

Electric quadrupole moment: $\vec{Q}^{(e)}_{\alpha\beta} = \frac{1}{2i\omega}\int [r_\alpha J_\beta + r_\beta J_\alpha - \frac{2}{3}(\vec{r} \cdot \vec{J})\delta_{\alpha\beta}] d^3 r$

Magnetic quadrupole moment: $\vec{Q}^{(m)}_{\alpha\beta} = \frac{1}{3c}\int [(\vec{r} \times \vec{J})_\alpha r_\beta + (\vec{r} \times \vec{J})_\beta r_\alpha] d^3 r$

where r is the distance vector from the origin to point (x, y, z) in a Cartesian coordinate, c is the speed of light, and α, $\beta = x, y, z$. The far-field scattering is characterized by electromagnetic radiation reemitted by each type of multipoles in a periodic arrangement under plane wave illumination. Figure S3 shows the calculated scattered power of the unit cell #5 in the single-rod supercell, the constituent single nanorod of IRU, and the replaced IRU element, respectively. We found when the IRU is formed by combing two low-AR nanorods in a front-back arrangement, the hybridized MD mode in IRU exhibits a much smaller redshift trend with a significant broadening in the spectral coverage. Similar effect can be observed for element #4 in IRU design as well.

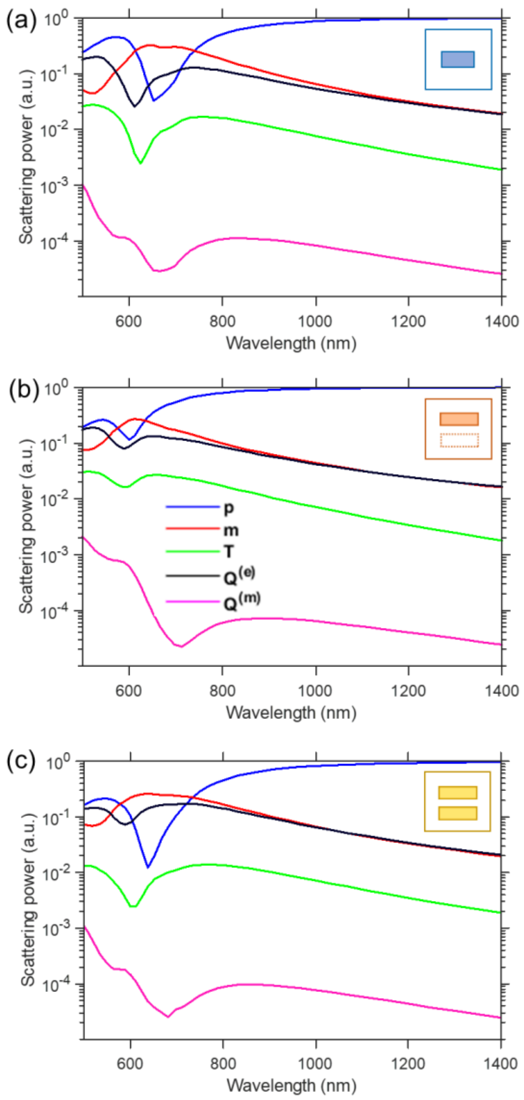

Figure S3. Calculated scattered power for individual electromagnetic multipoles of the element # 5 in **(a)** single-rod supercell, **(b)** the constituent single nanorod of IRU, and **(c)** the IRU supercell. The five strongest multipoles are presented including electric dipole **P**, magnetic dipole **m**, toroidal dipole **T**, electric quadrupole $\mathbf{Q}^{(e)}$, and magnetic quadrupole $\mathbf{Q}^{(m)}$.

Section S6: Theoretical performance of 0°/90° and 45°/135° PBS metagratings at the design wavelength of 730 nm.

For a beam deflector with a supercell length of Λ, the anomalous refraction or reflection angle (θ_r) upon the impinging of light with an incidence angle of θ_i at wavelength λ in free space is determined by the following equation[3],

$$\theta_r = \theta_i + \sin^{-1}(\lambda/\Lambda) \quad (1)$$

which corresponds to the first order of the diffraction angle of flat blazed gratings. Figure S4 shows the simulated electric-field distributions for 0°/90° and 45°/135° PBS metagratings, respectively, at λ = 730 nm. As we intentionally design two different periodicities of 240 nm and 320 nm for the unit cell of 0°/90° and 45°/135° PBS metagratings, respectively, the near-field distributions of the six-level phase gradient metasurfaces show anamalous reflected beams into ±30.46° (±22.34°) for 0°/90° (45°/135°) linear-polarized light, which agrees well with the predicted values calculated by the generalized Snell's laws.

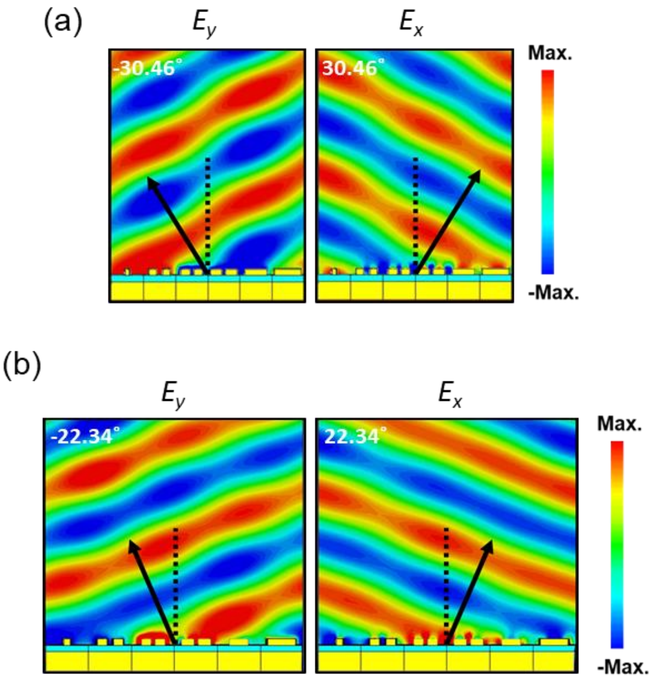

Figure S4. The simulated E_y (left) and E_x (right) field distributions for **(a)** 0°/90° and **(b)** 45°/135° PBS metagratings, respectively, at λ = 730 nm.

Section S7: The impacts of Ti-adhesion layers on the grating efficiency.

Figure S5 shows the simulated reflectance of a unit cell (element #1) when different thicknesses of Ti-adhesion layers above (t_{top}) and beneath (t_{bot}) the SiO_2 spacer are considered in the numerical model. Without the Ti layer, the reflectance is close to 0.9 (dashed line) and drops significantly for increasing Ti thickness. First, when comparing the cases either only the top (blue curve) or the bottom (red curve) Ti layer exists, the magnitude of efficiency drop is close between two cases when the thickness is below 5 nm, and a severer decay of efficiency takes place when t_{top} is larger than 5 nm compared to that of t_{bot}. Next, when both the top and bottom Ti-adhesion layer are considered, the variation of t_{top} under t_{bot} = 2 nm (yellow curve) show a similar slope to the blue curve with an offset of 7% efficiency drop, while the variation of t_{bot} under t_{top} of 3 nm (purple curve) show a similar slope to the red curve with an offset of 9% efficiency drop.

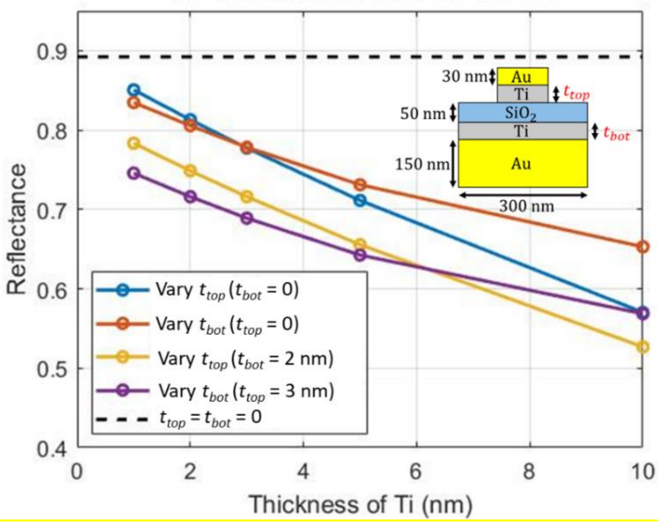

Figure S5. The simulated reflectance of a unit cell (element #1) when different thicknesses of Ti-adhesion layers above (t_{top}) and beneath (t_{bot}) the SiO_2 spacer are considered in the numerical model.

Section S8: Testbeds for the optical characterizations of the broadband PBS metagratings

Figure S6 shows the test layouts integrated for measuring the metagratings' spectral resolution, polarimetric contrast/modulation, and grating efficiency. A continuous-wave tunable laser system (procured from MSquared Lasers Inc.) was used as a light source. The laser beam has a linewidth of ~50 kHz (or 10–7 nm), which is ~7 orders of magnitude finer than the spectral resolving power of our NIR PBS metagratings, and the power stability is within 1% during the measurements of each metagrating sample. The laser beam passes through an achromatic waveplates and a rotatable wire grid polarizer to set the polarization state, attenuate light intensity (half-wave plate), and render the intensity out of the polarizer independent of the polarizer angle (quarter-wave plate). For the evaluating the metagratings' functionality of spectral dispersion, we used twin cameras (Figure S6a, Basler ace cA2040-55um, 6 MPixels; 7.4 × 5.2 mm sensor area) to record the 0°, 90°, 45°, 135° spectral, spatial, and polarimetric images. The agreement between measured and predicted spectral resolution is within 0.02 nm in the spectral region of interest. We note that the measured spectral resolution is ~ 0.02 nm coarser than the predicted value. This is probably due to imperfect catalog lenses or measurement uncertainty. The agreement is within one tenth of the design spectral resolution and can be accounted in the instrument calibration process, thus meeting the required performance of HiMAP-NIR. A power meter was used to measure the radiance diffracted by the metagrating ($\boldsymbol{I_d}$) and the radiance reflected by the gold substrate ($\boldsymbol{I_r}$) (Figure S6b and S6c). The diffraction efficiency is defined as the ratio of $\boldsymbol{I_d}$ divided $\boldsymbol{I_r}$. This definition is consistent with the previous studies (see reference 24 and 25 in main text).

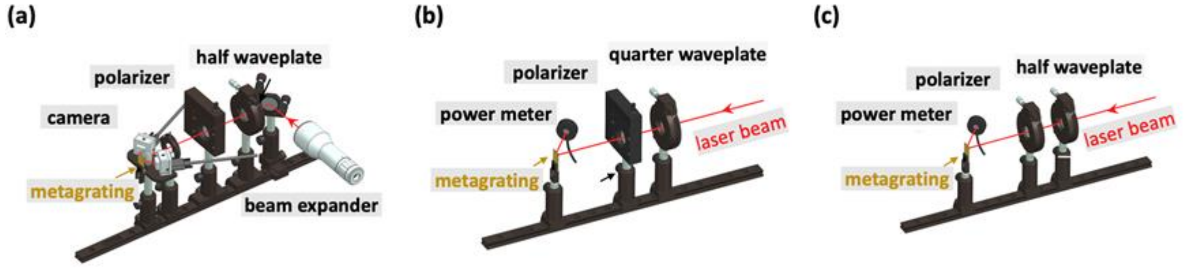

Figure S6. Illustration of the test setups to quantify the optical performance of the fabricated PBS metagratings including **(a)** the spectral resolution measurements, **(b)** the quantification of polarimetric modulation and polarization contrast, and **(c)** the grating efficiency measurements. The red line depicts the light path of the laser beam.

Section S9: Measurement setup for the spectral polarimetry images recorded using a fabricated NIR metagratings

To demonstrate the HiMAP-NIR measurement concept, we recorded the spatially, spectrally, and polarimetrically resolved images (Figure 5d in main text) by using the testbed shown in Figure S7. The white light source was first coupled to a beam expander, travel through an optical bandpass filter (transmissive from 680 to 780 nm) followed by a polarizer which sets the polarization states of the input beam, and shone on a variable line grating. The line grating target provides 18 spatial resolution settings spanning from 1.25-line pairs per millimeter (lp/mm) to 250 lp/mm. The measurements used the spatial resolution settings up to 16.67 lp/mm which is the setting closest to the maximum design performance (20 lp/mm) of the test bed. An aperture was placed in front the imaging lens to ensure an illuminating area of 5-mm diameter on the fabricated metagratings.

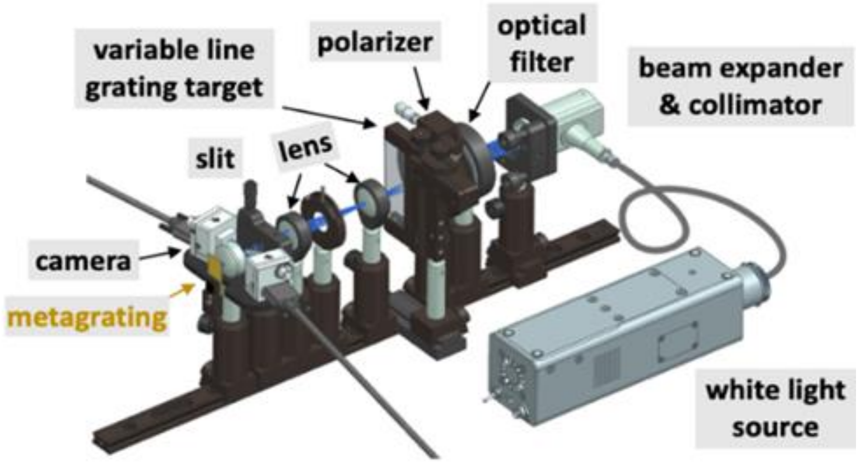

Figure S7. Illustration of the integrated testbed that records spectral polarimetry images under while light illumination.

Based on the generalized laws of refraction and reflection (Equation 1 of Section S6), the 0°/90°-polarized (45°/135°-polarized) diffraction patterns resided in the inner-pair (outer-pair) due to its longer (shorter) length of supercells. In term of spectral dispersion, longer wavelengths incident experience more angular dispersion as shown in Figure 5d in main text. In addition, by varying the polarized states of the incident white light source, the polarimetric modulation and polarization contrast can be observed. The recorded images demonstrate that the broadband PBS metagrating enables three key functionalities needed by the spaceborne HiMAP-NIR instrument: wide field-of-view imaging, simultaneous quantification of four linear polarized states, and spectral light dispersion.

References

[1] N. Papasimakis, V. A. Fedotov, V. Savinov, T. A. Raybould, N. I. Zheludev, *Nat. Mater.* **2016**, 15, 263.
[2] V. Savinov, V. A. Fedotov, N. I. Zheludev, *Physical Review B* **2014**, 89, 205112.
[3] N. Yu, P. Genevet, M. A. Kats, F. Aieta, J.-P. Tetienne, F. Capasso, Z. Gaburro, *Science* **2011**, 334, 333.
